# Prevalence of undernutrition and associated factors among children aged between six to fifty nine months in Bule Hora district, South Ethiopia

**DOI:** 10.1186/s12889-015-1370-9

**Published:** 2015-01-31

**Authors:** Mandefro Asfaw, Mekitie Wondaferash, Mohammed Taha, Lamessa Dube

**Affiliations:** Bule Hora District Health Office, Borena zone, Bule Hora, Ethiopia; Department of Population and Family Health, College of Public Health and Medical Sciences, Jimma University, Jimma, Ethiopia; Department of Epidemiology, College of Public Health and Medical Sciences, Jimma University, Jimma, Ethiopia

**Keywords:** Children, Undernutrition, Stunting, Wasting and underweight

## Abstract

**Background:**

More than one-third of deaths during the first five years of life are attributed to undernutrition, which are mostly preventable through economic development and public health measures. To alleviate this problem, it is necessary to determine the nature, magnitude and determinants of undernutrition. However, there is lack of evidence in agro-pastoralist communities like Bule Hora district. Therefore, this study assessed magnitude and factors associated with undernutrition in children who are 6–59 months of age in agro-pastoral community of Bule Hora District, South Ethiopia.

**Methods:**

A community based cross-sectional study design was used to assess the magnitude and factors associated with undernutrition in children between 6–59 months. A structured questionnaire was used to collect data from 796 children paired with their mothers. Anthropometric measurements and determinant factors were collected. SPSS version 16.0 statistical software was used for analysis. Bivariate and multivariate logistic regression analyses were conducted to identify factors associated to nutritional status of the children Statistical association was declared significant if p-value was less than 0.05.

**Results:**

Among study participants, 47.6%, 29.2% and 13.4% of them were stunted, underweight, and wasted respectively. Presence of diarrhea in the past two weeks, male sex, uneducated fathers and > 4 children ever born to a mother were significantly associated with being underweight. Presence of diarrhea in the past two weeks, male sex and pre–lacteal feeding were significantly associated with stunting. Similarly, presence of diarrhea in the past two weeks, age at complementary feed was started and not using family planning methods were associated to wasting.

**Conclusion:**

Undernutrition is very common in under-five children of Bule Hora district. Factors associated to nutritional status of children in agro-pastoralist are similar to the agrarian community. Diarrheal morbidity was associated with all forms of Protein energy malnutrition. Family planning utilization decreases the risk of stunting and underweight. Feeding practices (pre-lacteal feeding and complementary feeding practice) were also related to undernutrition. Thus, nutritional intervention program in Bule Hora district in Ethiopia should focus on these factors.

## Background

Undernutrition among children is a major public health problem in developing countries including Ethiopia [1-3]. Globally, children with moderate and severe acute undernutrition are approximately 60 million and 13 million respectively [1,2]. Between 8 to 11 million under-five children die each year globally [2,4]. More than 35% of these deaths are attributed to undernutrition, which are mostly preventable through economic development and public health measures [3]. Undernutrition among children is a critical problem because its effects are long lasting and go beyond childhood. It has both short and long term consequences [1,5]. For instance, undernourished as compared to non-undernourished children are physically, emotionally and intellectually less productive and suffer more from chronic illnesses and disabilities [6-9].

Although Ethiopia has already achieved a remarkable progress in reducing under-five mortality in the last decades [10,11], undernutrition among children is still a common problem in this country [10-13]. Ethiopia is one of the countries with very high burden of undernutrition [11]. Undernutrition can best be described in the country as a long term year round phenomenon due to chronic inadequacies in food combined with high levels of illness in under-five children. It is the underlying cause of 57% of child deaths. Undernutrition is currently the most widespread and serious health problem of children. According to Ethiopian DHS 2011 data, among under-five children 44%, 29% and 10% were stunted; underweight and wasted respectively [10].

Causes of undernutrition are numerous and multifaceted. Undernutrition among children depends on complex interactions of various factors like: socio-demographic, environmental, reproductive, institutional, cultural, political and regional factors [3,14,15]. Already, many studies have been conducted to find the magnitude and predictors of undernutrition in Ethiopia and elsewhere [16-28].

Studies done in different parts of Ethiopia consistently reported high prevalence of undernutrition in under-five children [12,16,17,19,23,27,28]. Past studies have also shown that economic status [17,19,27], place of residence [17,18], education of the mother [12,18,27], age of the mother [13,18,28], occupation of the mother [18], source of water [17,18,27], availability of latrine [17], child morbidity [27], sex of the child [17,18,27] age of the child [12,19,27], method of feeding [12,27], age of initiation of complementary feeding [12,19], birth interval of the child [17,18], total number of children ever born to the mother [27] and maternal nutritional status [17,18] were factors associated with undernutrition among under-five children.

Majority of the studies conducted in Ethiopia have been on agrarian communities and urban dwellers. Only two studies [27,28] have been done in agro-pastoralist communities of Ethiopia. These two studies [27,28] had emphasized more on household characteristics, maternal and child characteristics and economic variables associated with child undernutrition. Furthermore, the studies overlooked important variables like child care practice, child history of illness, and family planning utilization. Therefore, there is a knowledge gap on comprehensive understanding of magnitude and determinants of undernutrition in agro-pastoralist community. Therefore, this study was aimed to bridge the above mentioned knowledge gap by assessing the prevalence and associated factors of undernutrition in agro-pastoralist community, Bule Hora district, Southern Ethiopia. The district is one of the most underserved areas in terms of access to essential services and characterized by a high level of child undernutrition, food insecurity and drought [29].

Therefore, determining the magnitude and identifying the risk factors for child undernutrition in the study area is important to guide public health planners, policy makers and implementers to plan and design appropriate intervention strategies in order to enhance nutritional status of children.

## Methods

### Study setting

The study was conducted in Bule Hora district, one of 14 districts in Borana Zone. The Borana zone is one of 13 administrative zones within Ethiopia’s Oromia State. It is located in the Southern part of the state (between 3°36 – 6°38’ North latitude and 3°43’- 39°30’ East longitude) and borders Kenya. Yabello is the capital town of the Borana zone and lies 570 km south of Addis Ababa (capital city of Ethiopia). Eighty percent of population lives in rural areas. The urban population is involved in private business, government employment or farming. The agricultural sector is very important in the rural areas of the Borona zone. In the highlands, people predominantly grow (cash) crops with some livestock for additional income. The people in the lowlands keep livestock as major economic activity, based on traditional pastoralist systems. The poor quality of drinking water results in serious health problems in the zone. Water-related diseases, such as cholera and diarrhoea, are among the major causes of child mortality and morbidity. Child mortality rate for the Borana Zone is 142 per 1000 live births. Drought is a common phenomenon in the area [29]. Bule Hora district has 48 (43 rural and 5 town) kebeles (smallest administrative units) with a total population of 317,341 of which 52,044 are under five children as estimated in 2007 census. The total households in the district were 66, 113 [30].

### Study design

A community based cross-sectional study was conducted in Bule Hora District from February 25 to March 25, 2013.

### Study participants

The source population of this study included all 6–59 months old children (paired with their mothers) who lived in the district. The study population were the randomly selected 6–59 months old children (paired with their mothers) who lived at least for six months in the district.

### Sample size and sampling technique

The sample size was calculated using a formula for estimation of a single proportion according to the following assumptions: 44% of under-five children are stunted (P) [10], with 95% confidence interval and 5% marginal of error (d). The calculated sample size was 379. As a multistage sampling technique was employed to identify study subjects, a design effect of 2 was used. Also 5% was added for non-responses. Thus, the final sample size was 796.

A multistage stratified sampling technique was used to identify study subjects after the kebeles were stratified into urban and rural kebeles. Out of 48 kebeles in the district, eight (2 urban and 6 rural) were selected by lottery method. In these selected kebeles, 10,960 children who are 6–59 months were identified with their respective households using the registration at health posts and the community based nutrition registration. The calculated sample (796) was proportionally allocated to the selected kebeles based on the total number of households with 6–59 months children in each kebele and study subjects were identified by simple random sampling of the households. One child was selected by lottery method when more than one child was present in selected households.

### Measurements

The main outcome variable in this study was nutrition status measured as underweight, stunting, and wasting. Independent variables include: socio-demographic variables (age of child and mother, child sex, family size, income, maternal/paternal education and occupation, marital status of the mother and number of livestock owned); child characteristics (birth order, and childhood illness); and child caring practices (exclusive breast feeding, hygiene, health care seeking and immunization). Maternal characteristics (number of children ever born, antenatal care visits, health status during pregnancy, use of extra food during pregnancy and autonomy in decision-making); and environmental health condition (water supply, sanitation and housing conditions) were also independent variables in this study.

Anthropometric data were collected through measurement of length/height and weight of all children. Weight was measured with minimum clothing and no shoes using a hanging scale and electronic beam balance in kilogram for those 6–24 months and 24–59 months respectively, to the nearest of 0.1 kg. Measurement of length was done in a lying position with wooden board for children of age under two years and measurement of height for children above two years stature was measured in a standing position in centimeters to the nearest of 1 cm. WHO Anthro version 3.2.2 software was used to convert the anthropometric measures; weight, height/length and age values into Z-scores of the indices; Height-for-Age(HAZ), Weight-for-Height(WHZ) and Weight-for-Age(WAZ) taking sex into consideration using WHO 2006 standards [9].

A low height-for-age, below -2SD of the reference population, indicates stunting while below -3SD indicates severe stunting. A low weight-for-height, below -2SD of the reference population, indicates wasting, while below −3SD indicates severe wasting. A child with a weight-for-age z-score below -2SD of the reference population is underweight while a child below or -3SD is severely underweight [9,23].

Mid Upper Arm Circumference (MUAC) was measured using non-stretchable tape on left mid upper arm to the nearest 1 mm. MUAC below 12.5 cm indicates acute undernutrition. Edema was assessed by applying medium thumb pressure on upper side of both feet for three seconds. It was diagnosed if a bilateral depression (pitting) remained after the pressure is released.

The socio-demographic variables were adopted from the women and household questionnaires of the EDHS [9]. Education status was measured according to the education levels in Ethiopia: no formal education, having a primary level education, secondary level and above. Finally, Education status was divided into two levels: had formal education and had no formal education. To assess the childhood illness, the mothers were asked whether their children had been affected by diarrhea in the past two weeks. Diarrhea is defined as having three or more loose or watery stool in a 24-hour’s period the two weeks period prior to the survey [31]. Pre-lacteal feeding was assessed by asking a mother whether the child had been given anything to drink other than breast milk in the first three days after delivery. Exclusive breast feeding was assessed by asking a mother for how long the child fed breast exclusively.

Data were collected using structured questionnaire via face to face interview and anthropometric measurements. The questionnaire was initially prepared in English after reviewing literature by Investigators and then translated into Afan Oromo/Amharic, then retranslated to English to check the equivalence.

Eight nurses and eight community health workers from each selected kebeles were involved in the data collection. Two days’ training was provided to data collectors and supervisors. Pretest was done on 5% of sample size in non selected kebeles and some modifications were made accordingly. Data was collected under the supervision of the principal investigator. Incomplete questionnaires were completed by making second visits to the homes. Weighing scales were calibrated with known weight object regularly. The scales indicator was checked against zero reading after weighing every child.

### Analysis

Statistical analyses were done using SPSS 16.0 statistical software. The data were exported to SPSS after the data were first coded and then entered in the computer using Epi-Data version 3.1 Software. Descriptive statistics like frequencies or proportions were first done and presented by tables. Then bivariate analysis was done for the three outcome variables: stunting, underweight and wasting separately. The independent variables with p-value less than 0.25 during bivariate analysis were selected as candidate for multivariable analysis. Multivariable binary logistic regressions were fitted by using backward elimination technique to identify determinants of stunting, wasting and underweight separately. Association between dependent and independent variables was assessed using OR and 95% Confidence Interval (CI). Statistical association was declared significant if p-value was less than 0.05. Multicollinearity between independent variables were checked and found not present.

### Ethical consideration

The ethical clearance was obtained from Jimma University, Ethical Review Board. Written informed consent was also obtained from each respondent. Participants with diarrhea, respiratory tract infections and undernutrition were referred to health institutions and organizations working on nutrition.

## Results

From the total of 796 planned study participants, complete response was obtained for 778 (97.7%). Characteristics of the sampled children, their mothers and their households are presented in Table 1. Among the total respondents, 714 (91.8%) were married. Nearly half of the households had two under five children. The mean age was 28 (standard deviation = 14.6) months for both sex. The average total number of children born to a mother was 4.2 children (Standard Deviation = 2.5).

**Table 1:** **Socio-demographic characteristics of the sampled children and their mothers, Bule Hora district, 2013 (n = 778)**

**Variables**		**Frequency (%)**
Ethnicity	Oromo	696(89.5)
	Gedeo	50(6.4)
	Gurage	14(1.8)
	Amhara	11(1.4)
	Burji and Koyira	7(0.9)
Marital status	Married	714(91.8)
	Divorced	25(3.2)
	Widowed	33(4.2)
	Single	6(0.8)
Maternal education	Illiterate	497(63.9)
	Primary	239(30.7)
	Secondary	34(4.4)
	Certificate & Diploma	8(1.1)
Maternal age (in years)	15-19	10(1.3)
	20-24	73(9.4)
	25-29	262(33.7)
	30-34	190(24.4)
	35-39	148(19.0)
	40-44	86(11.1)
	>44	9(1.2)
Family size	2-5	350(45)
	>5	428(55)
Numbers of under-children five in HH	1	359(46.1)
	2	373(47.9)
	3-4	46(6)
Child sex	Male	384(49.4)
	Female	394(50.6)
Child age (in months)	6-11	123(15.8)
	12-23	189(24.3)
	24-35	160(20.6)
	36-47	188(24.2)
	48-59	118(15.2)
Child birth order	1	151(19.4)
	2-4	361(46.4)
	>4	266(34.2)

Among the total participants, 704 (90.5%) were exclusively breastfed for four to six months, 94 (12.1%) were given pre-lacteal feeding. Water, cattle milk and butter were the most commonly used foodstuffs for pre-lacteal feeding. Concerning vaccination, 16 (2.1%) of the children did not receive any form of vaccine and 414 (54.3%) were fully vaccinated. The most frequent child health problem was diarrhea. Three hundred seventy seven (48.7%) and 195 (25.1%) children had diarrhea in the past 12 months and 2 weeks prior to data collection respectively.

From the total mothers of children interviewed, 214 (27.5%) of them did not attend ANC service during their pregnancy. Only one-tenth of the children were born in health institutions. Among the total mothers of children interviewed, about 723 (92.9%) had information about family planning methods but only 360 (49.8%) of them had ever used family planning method (Table 2).

**Table 2:** **Child caring practices and maternal health service utilization, Bule Hora district, 2013 (n = 778)**

**Variables**		**Frequency (%)**
Exclusive Breastfeeding (in months)	<4	32(4.1)
	4-6	704(23.0)
	>6	34(4.4)
	Unknown	8(1.0)
Vaccination status	Not vaccinated	16(2.1)
	Partially vaccinated	348(45.7)
	Fully vaccinated	414(54.3)
Vitamin A supplementation	Yes	401(51.5)
	No	377(48.5)
Fed Pre-lacteal feeding	Yes	94(12.1)
	No	684(87.9)
Place of delivery	Home	693(89.1)
	Health facility	85(10.9)
Diarrheal morbidity in the past year	Yes	377(48.5)
	No	401(51.5)
Diarrheal morbidity in the last two weeks	Yes	195(25.1)
	No	583(74.9)
Episodes of diarrheal morbidity per year*	1 episodes	268(71.1)
	2 episodes	84(22.3)
	≥3 episodes	25(6.6)
Child ever taken to Health institutions	Yes	522(67.1)
	No	256(32.9)
Total number of children born to a mother	≤4	483(62.1)
	>4	295(37.9)
Antenatal care follow-up	Yes	564(72.5)
	No	214(27.5)
Knowing FP methods	Yes	723(92.9)
	No	55(7.1)
Ever used of FP methods	Yes	360(49.8)
	No	363(50.2)
Extra meal during pregnancy/lactation	Yes	513(65.9)
	No	265(34.1)

FP- family planning methods.

*Total may not be equal to 778.

### Prevalence of undernutrition

Prevalence of stunting, underweight and wasting among study participants were 47.6%, 29.1% and 13.4% respectively. Prevalence of severe stunting, underweight and wasting among the children were 20.2%, 6% and 3.9% respectively. MUAC measurement also indicated that 10% of the children were undernourished (<12.5 cm) (Table 3).

**Table 3:** **Proportion of undernutrition among children aged between six to fifty nine months, Bule Hora district, 2013 (n = 778)**

**Undernutrition indicators**	**Frequency (%)**
Normal HAZ (≥ −2SD)	408(52.4)
Moderate stunting (HAZ ≥ −3SD & < −2SD)	213(27.4)
Severe stunting (HAZ < −3SD)	157(20.2)
Normal WAZ (≥ −2SD)	551(70.8)
Moderate underweight (WAZ ≥ −3SD & < −2SD)	108(23.1)
Severe underweight (WAZ < −3SD)	47(6.0)
Normal WHZ (≥ −2SD)	674(88.6)
Moderate Wasting (WHZ ≥ −3SD & < −2SD)	74(9.5)
Severe wasting (WHZ < −3SD)	30(3.9)
Moderate acute undernutrition (MUAC ≥ 11.5 cm & < 12.5 cm)	60(7.7)
Severe acute undernutrition (MUAC < 11.5 cm)	18(2.3)
Acute undernutrition (MUAC < 12.5)	78(10)

### Determinants of undernutrition

Results of the logistic regression of underweight are shown in Table 4. Male children were 2.5 times (AOR=, 95% CI: 1.5-4.1) more likely to be underweight than female children. Children having diarrhea in the past two weeks prior to the data collection were 3.9 times (AOR = 3.9, 95% CI: 2.2-6.8) more likely to develop underweight than children without diarrheal disease. Risk of underweight among children whose fathers were illiterate was 6.7 times (AOR = 6.7, 95% CI: 1.8-62.2) more likely than children whose fathers were literate.

**Table 4:** **Factors associated with underweight among children aged between six to fifty nine months, Bule Hora district, 2013 (n = 778)**

**Variables**		**Underweight**		**Crude OR (95% CI)**	**Adjusted OR (95% CI)**
		**Yes**	**No**		
Child sex	Female	82	312	1.0	1.0
	Male	145	239	2.3(1.6-3.1)*	2.5(1.5-4.1)*
Total number of children born to a mother	≤4	372	111	1.0	1.0
	>4	179	116	2.2(1.6-3.0)*	1.8(1.1-3.1)*
Diarrheal morbidity in the last two weeks	No	125	458	1.0	1.0
	Yes	102	93	4.0(2.9-5.7)*	3.9(2.2-6.8)*
Paternal formal education	Yes	106	193	1.0	1.0
	No	121	358	2.1(1.5-2.9)*	6.9(1.7-62.2)*
Availability of latrine	Yes	162	461	1.0	1.0
	No	65	90	2.05(1.4-3.0)*	1.2(0.72-1.9)
Maternal formal education	No	175	322	1.0	1.0
	Yes	52	229	2.4(1.7-3.4)*	1.1(0.35-3.4)
Fed Pre-lactation feeding	No	188	496	1.0	1.0
	Yes	39	55	1.87(1.2-2.9)*	1.1(0.63-1.9)

*Significant at p-value < 0.05.

Regarding stunting, male children were 2.8 times (AOR = 2.8, 95% CI: 1.5-5.3) more likely to be stunted compared with female children. Children who had a diarrhea in the past two weeks prior to the data collection were 2.5 times (AOR = 2.5, 95% CI: 1.2-5.3) more likely to be stunted than children without diarrheal disease. Children who received pre-lacteal feeding were 3.8 times (AOR = 3.8, 95% CI: 1.2-12.2) more likely to be stunted than children who did not receive pre-lacteal feeding at the time of birth (Table 5).

**Table 5:** **Factors associated with stunting among children aged between six to fifty nine months, Bule Hora district, 2013 (n = 778)**

**Variables**		**Stunted**		**Crude OR (95% CI)**	**Adjusted OR (95% CI)**
		**Yes**	**No**		
Child sex	Female	164	230	1.0	1.0
	Male	206	178	1.62(1.2-2.2)*	2.8(1.5-5.3)*
Diarrheal morbidity in the last two weeks	No	253	330	1.0	1.0
	Yes	117	78	2.0(1.4-2.7)*	2.5(1.2-5.3)*
Maternal formal education	Yes	106	175	1.0	1.0
	No	264	233	1.9(1.4-2.5)*	1.3(0.6-2.5)
Attended ANC	Yes	240	324	1.0	1.0
	No	130	84	2.1(1.5-2.9)*	1.3(0.8-2.0)
Ever used of FP	Yes	136	224	1.0	1.0
	No	211	152	2.3(1.7-3.1)*	1.5(1.0-2.1)
Fed Pre-lacteal feeding	Yes	321	363	1.0	1.0
	No	49	45	1.2(0.80-1.9)*	3.8(1.2-12.2)*
Had extra meal during pregnancy or lactation	Yes	209	304	1.0	1.0
	No	161	104	2.3(1.7-3.1)*	0.65(0.4-0.9)
Availability of latrine	Yes	279	344	1.0	1.0
	No	91	64	1.8(1.2-2.5)*	0.89(0.5-1.4)
Amount of water used by household per day (liter)	<20	9	6	1.0	1.0
	20-40	282	353	0.5(0.18-1.5)	7.15(0.5-92.5)
	>40	79	49	1(0.36-3.2)	2.6(0.96-6.8)
Total family size	≤5	138	212	1.0	1.0
	>5	232	196	1.8(1.4-2.4)*	1.25(0.8-2.0)

ANC- Antenatal care, FP- Family Planning methods.

*Significant at p-value <0.05.

Regarding wasting, children who had a diarrhea in the past two weeks prior to the data collection were 2.7 times (AOR = 2.7, 95% CI: 1.1-6.4) more likely to be wasted than children had no diarrhea. It was also observed that the likelihood of being wasted was significantly higher for children who started complementary feeding before the age of 6 months. As compared with children who started complementary feeding at 6 months, the risk of wasting was 3.3 times (AOR = 3.3, 95% CI: 1.5-7.4) more for children who started complementary feeding before 6 months. Children whose mothers had never used family planning were 3.8 times (AOR = 3.8, 95% CI: 1.3-4.4) more likely to be wasted compared to children whose mother had ever used family planning (Table 6).

**Table 6:** **Factors associated with wasting among children of 6–59 months of age, Bule Hora district, 2013**

**Variables**		**Wasting**		**Crude OR (95% CI)**	**Adjusted OR (95% CI)**
		**Yes**	**No**		
Child sex	Female	39	356	1.0	1.0
	Male	65	318	1.8(1.2-2.8)*	1.78(0.8-2.8)
Maternal formal education	Yes	27	254	1.0	1.0
	No	78	419	1.8(1.1-2.9)*	1.5(0.9-2.6)
Diarrhea in the last two weeks	No	49	534	1.0	1.0
	Yes	55	140	4.2(2.8-6.5)*	2.7(1.1-6.4)*
Age at complementary food started (in months)	≥6	59	498	1.0	1.0
	<6	46	175	2.3(1.5-5.8)*	3.3(1.5-7.4)*
Ever use of FP	Yes	41	318	1.0	1.0
	No	52	311	1.3(0.84-2.0)	3.8(1.3-11.6)*
Availability of latrine	No	73	550	1.0	1.0
	Yes	31	124	1.9(1.2-3.0)*	0.61(0.38-0.99)
Availability of window	Yes	62	516	1.0	1.0
	No	42	158	2.2(1.4-3.4)*	0.62(0.38-1.0)
Providing Pre-lacteal feeding	No	86	598	1.0	1.0
	Yes	18	76	1.6(0.94-2.9)*	1.28(0.71-2.3)

FP- Family Planning methods.

*Significant at p-value <0.05.

## Discussions

Prevalence of child undernutrition was high in the district. This study noted that diarrheal morbidity, child sex, fathers’ educational status, number children ever born to a mother, pre–lacteal feeding, age at initiation of complementary feed and family planning methods use were significantly associated with undernutrition.

This study showed that prevalence of child undernutrition; stunting and wasting were higher in the district in comparison with the regional prevalence reported by EDHS 2011 (41.4% stunting and 9.7% wasting) [11]. The discrepancy might be due to small sample size compared to that of national data and the exclusion of children under the age of six months. Prevalence of stunting in the district (47.6%) was very high compared with results of study done in a pastoralist community of Ethiopia which was 34.4%; however, the prevalence of wasting and underweight were lower [27]. There was high prevalence of wasting (13.4%) in the study area alarming to increased risk of death to children. It signifies acute nutritional problem probably due to illness like diarrheal morbidity and/or food shortage. High chronic undernutrition also signifies children’s failure to grow; impact on both physical and mental capacity of the affected children.

Parental educational status was independently associated with children’s underweight. Children of uneducated father were more likely to be underweight when compared with children of educated fathers. This is consistent with study conducted in Ethiopia and Bangladesh [24,25], which showed children from uneducated fathers were positively associated with child undernutrition. It is argued that fathers with higher educational status in the society have the ability to make decisions that improve the nutritional status of children while those with low educational status do not [24,25].

Infections play a major role in the etiology of undernutrition because they result in increased needs and high energy expenditure, lower appetite, nutrient losses due to vomiting, diarrhea, poor digestion, mal-absorption and the utilization of nutrients and disruption of metabolic equilibrium [14]. In this study, presence of diarrheal morbidity in the last two weeks prior to data collection was the contributing factor for stunting, underweight and stunting. The results of this study are in agreement with results of studies conducted in Ethiopia and Vietnam [12,22]. The association between diarrheal morbidity 2 weeks prior to data collection and stunting (a chronic form of undernutrition) was because the diarrheal morbidity of 2 weeks period was a proxy of diarrheal morbidity in earlier life.

Compared to girls, the likelihood of both stunting and underweight was higher among boys. Similarly, many studies in Ethiopia and elsewhere have reported that under-five male children are more likely to become stunted than their female counterparts [12,16,22,23,27]. This could be because boys are more influenced by environmental stress than girls. Thus, boys are more likely to display impact of chronic undernutrition; especially in environments where stresses are at play, like repeated infections and exposure to toxins and air pollutants [22,23]. Studies done in Ethiopia and Vietnam also reported that boys were more likely to be under-weight [22,17].

Pre-lacteal feeding was also a risk factor for being stunted. This study showed that children who fed pre-lacteal feeding were more likely to be stunted. This study is consistent with a study conducted in agrarian community of Ethiopia [12]. The higher risk of stunting among children who fed pre-lacteal feeding might be due to its negative impact on breastfeeding. When children are not breastfed appropriately, they are at higher risk of under-nourishment [12].

This study showed that there is a negative relationship between mother’s use of family planning methods and nutritional status (stunting and wasting) of children. This study was in line with the report of Ethiopian DHS 2011, which shows that there is an inverse relationship between the length of the preceding birth interval and the proportion of children who are stunted. The longer the interval, the less likely it is that the child will be stunted [10,19]. The probable reason is that when there are too many children who are closely spaced in the family, there may be the tendency for undernutrition to occur.

This study also identified that children born to a mother who gave birth to more than four children were more likely to be underweight when compared to children from a mother who gave birth to less than four children. This finding is in line with the study conducted in Vietnam and Bangladesh [24,25]. This could be because families with more children experience more economic strain for food consumption and hence they are more likely to suffer from poor nutritional status. In other words, inadequate allocation of household resources among many children may lead to the low nutritional status. Particularly, poor families cannot fulfill the nutritional requirements of the children. Families with more children generally devote less time to take care of their children [28].

Age at initiation of complementary feed was also a significantly associated with being wasted. Children who started complementary feeding before the age of six months were more likely to be wasted when compared with the children who started at the age of six months. This study was in line with the study conducted in Vietnam and Ludhiana which showed that children who exclusively breast fed for <6 months were more likely to wasted when compared with those exclusively breast fed for ≥6 months [22,26].

This study has accomplished its objectives to determine the prevalence and factors associated with stunting, wasting and underweight among children aged 6–59 months in agro-pastoralist community of Bule Hora District, South Ethiopia. However, there are some limitations. First, since it was a cross-sectional design, it was difficult to examine any potential temporal relationships. Second, there is potential recall bias among respondents answering questions relating to events happening in the past, such as the child’s history of illness and breastfeeding patterns immediately after birth and then after. Finally, information on some important confounding variables such as parasitic infection, HIV status, mother’s pre-pregnancy weight, the child’s birth weight and the daily caloric intake were not collected which could cause problems in interpreting the results. Parasites can affect the nutrition and growth of children by depriving them of nutrients, impairing intestinal absorption of fat, nitrogen and vitamin A, and reducing food intake [32]. Also information on the mother’s size and pre-pregnancy weight and the child’s birth weight, all of which are strongly associated with the child’s size are lacking. The exact daily caloric intake was also not ascertained, nor was HIV status. The household wealth index and household food insecurity were also not assessed.

## Conclusions

In conclusion, there are high prevalence rates of undernutrition (stunting, wasting and underweight) among the under five children in agro-pastoralist communities. Factors associated to nutritional status of children in agro-pastoralist communities are similar to the agrarian communities. Being male, diarrheal morbidity and pre-lacteal feeding were associated with increased risk of stunting. Being male, number of children ever born to a mother greater than four, diarrheal morbidity and illiterate father were significantly associated with underweight. Diarrheal in the last two weeks, complimentary feeding before 6 months after birth and not using family planning methods were associated with wasting. Therefore, prevention and control of diarrheal disease through improving access to safe and adequate water supply, vaccination, housing, sanitation and hygiene practices should be considered. Intervention should focus on improving promotion of nutrition education, promotion of better child care and utilization of family planning. Further research on dietary assessment is required.
